# Junction opener enables CAR T cell treatment of solid tumors

**DOI:** 10.1038/s41598-026-43093-8

**Published:** 2026-03-07

**Authors:** Steven J. Reed, Shibbu Sharma, Carrie Novák, Jiho Kim, Kaman Kim, Erene Niemi, Nicholas Korjeff, André Lieber, Steven G. Reed, Malcolm S. Duthie, Darrick Carter

**Affiliations:** 1 HDT Bio, 1150 Eastlake Ave E, Suite 200A, Seattle, WA 98109 USA; 2 PAI Life Sciences Inc, 1551 Eastlake Ave E, Suite 250, Seattle, WA 98102 USA; 3 Xyphos Biosciences, an Astellas Company, 480 Forbes Blvd, South San Francisco, CA 94080 USA; 4 Division of Medical Genetics, School of Medicine, University of Washington, Seattle, WA 98195 USA

**Keywords:** CAR T cells, Infiltration, Tumor microenvironment, Junction opener, Solid tumors, Cancer immunotherapy, Cancer therapeutic resistance

## Abstract

Physical barriers presented by solid tumors limit CAR T cell efficacy. Thus, technologies to increase CAR T cell access to solid tumors could have massive implications in advancing CAR T cell therapies. Here we demonstrate that treatment of solid tumor-bearing mice with our Junction Opener technology and CAR T cells enhances tumor control - potentially enabling CAR T cell immunotherapy for solid tumors.

**Supplementary Information:**

The online version contains supplementary material available at 10.1038/s41598-026-43093-8.

## Introduction

T cells expressing chimeric antigen receptors (CAR T) that target specific cancer antigens have revolutionized immunotherapy for hematological malignancies^1^. However, no CAR T cell products are approved for the treatment of solid tumors, a category that accounts for over 90% of all cancers.

Physical barriers inhibit infiltration into solid tumors, limiting CAR T cell efficacy. A key feature of epithelial tumors is the presence of intercellular junctions that link cells and impede penetration of many molecules^2–4^. Desmoglein 2 (DSG2) is a transmembrane adhesion glycoprotein with an important role in junction stability^5,6^. Elevated DSG2 expression has been demonstrated in numerous cancers including non-small cell lung cancer, skin carcinomas, cervical cancers, and colon adenocarcinomas^7–10^. DSG2 upregulation has been associated with resistance to treatment, indicating a tumor-promoting role across various tumor types^11^.

Adenovirus type 3 (Ad3) binding of DSG2 on epithelial cells triggers events reminiscent of epithelial-to-mesenchymal transition, with transient opening of intercellular junctions and improved access to receptors such as the CAR antigen HER2/neu^12–14^. Derived from the C-terminal knob domain of Ad3 capsids, the Junction Opener (JO) series of recombinant proteins bind to DSG2 and preferentially open tumor tight junctions^15^. The JO protein has been extensively characterized - including determining the crystal structure of the active trimeric form that represents the minimal configuration with measurable affinity for DSG2^16^. For junction-opening activity to permit penetrance, higher-order multimers are required and additional affinity maturation culminated in JO-4 that has a nearly 1000-fold increase in affinity for DSG2^16^. Binding of JO-4 leads to DSG2 shedding and transient tight junction opening. JO-4 treatment was well tolerated in non-human primates and shows significantly greater bioaccumulation in solid tumors compared to tissues expressing DSG2 under homeostatic conditions^17^. We hypothesized that JO-4 treatment would augment CAR T cell activity against solid tumors.

## Results

**Fig. 1:**
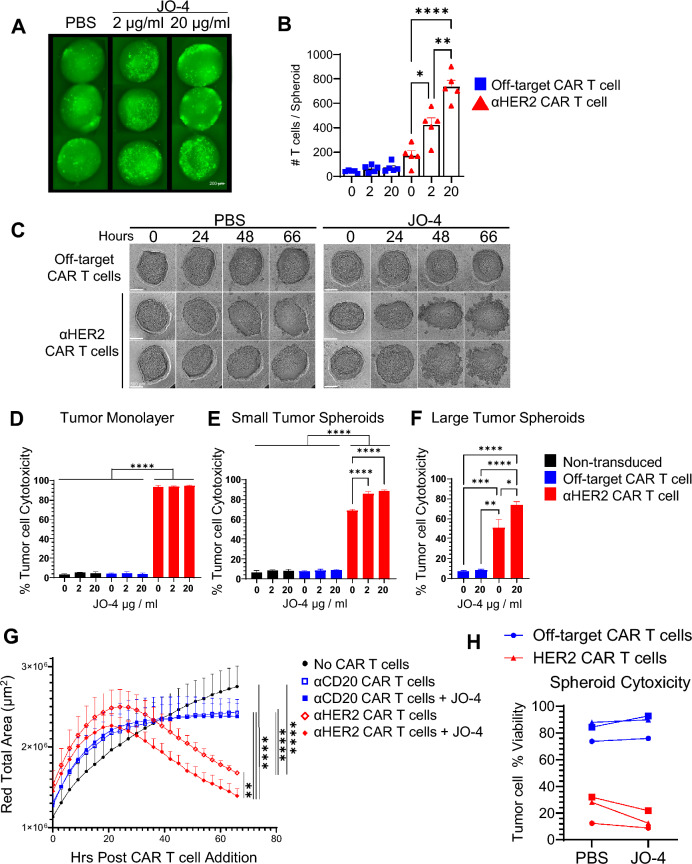
JO-4 treatment augments CAR T cell infiltration, antigen-specific disruption, and killing of tumor spheroids. BT-474 tumor spheroids were treated with JO-4, or PBS (vehicle). One-hour later, off-target, or αHER2 CAR T cells were added at a 4:1 effector:tumor ratio. (**A**) Tumor spheroid images 24hrs post-addition of eGFP-expressing αHER2-CAR T cells. (**B**) Quantification of tumor spheroid infiltrating CAR T cells. (**C**) Wide-field images of tumor spheroid-CAR T cell co-cultures. (**D**-**H**) BT-474 tumor cell (**D**) monolayers, (**E**, **G**-**H**) small tumor spheroids (~40 μm diameter), (**F**) large tumor spheroids (~1000 μm diameter), were pre-treated with either 0 (PBS), 2, or 20 μg/ml JO-4 for 4 hours. Non-transduced, off-target CAR, or αHER2 CAR, T cells were added at a 2.5:1 CAR T:tumor cell ratio (or matched total number of non-transduced T cells). **(G)** Representative tumor spheroid cytotoxicity over time. (**H**) Tumor viability at 68hrs. Each shape represents an individual donor (n=3). Bar graphs show mean ± SEM. Statistics: * = p<0.05, ** = p< 0.01, *** = p <0.001, **** = p<0.0001 as determined by student’s T test, or one-way ANOVA and Tukey’s multiple comparisons test.

To investigate the impact of JO-4 on CAR T cell therapy of solid tumors, the human mammary carcinoma BT-474 cell line was selected due to high expression of both a relevant CAR antigen (HER2) and DSG2. We utilized an *in vitro* BT-474 tumor spheroid model that forms intercellular tight junctions, treating them with either PBS or JO-4 prior to addition of either non-transduced, off-target (αCD20), or on-target αHER2 CAR T cells. JO-4 pretreatment permitted a significant increase in HER2 specific, but not off-target, CAR T cell infiltration of tumor spheroids (Fig. 1A, B). The magnitude of CAR T cell penetrance was associated with disruption of the spheroid and occurred in an antigen-specific manner with no spheroid disruption following incubation with off-target CAR T cells (Fig. 1C).

To assess the impact of JO-4 treatment on CAR T cell tumor cell killing of monolayers lacking DSG2 intercellular tight junctions, or tumor spheroids, tumor cells were pre-treated with JO-4 prior to the addition of non-transduced, off-target, or αHER2, CAR T cells. Minimal tumor cell killing was observed by non-transduced or off-target CAR T cells, in contrast to significant tumor cell killing by αHER2 CAR T cells (Fig. 1D-F). Within each T cell population, JO-4 treatment did not impact tumor monolayer killing suggesting that JO-4 treatment does not directly impact tumor cell viability or CAR T cell function (Fig. 1D). However, JO-4 treatment increased CAR T cell-mediated spheroid killing in a dose-dependent manner (Fig. 1E-F), reflective of CAR T cell infiltration and spheroid disruption (Fig. 1A-C). Real-time cytotoxicity assays corroborate these data, where JO-4 pre-treatment of spheroids co-cultured with αHER2 CAR T cells significantly increased spheroid killing but did not impact off-target CAR T cell spheroid cytotoxicity (Fig. 1 G, H). Together, these data indicate that JO-4 disruption of tight junctions permits CAR T cell penetrance into tumor spheroids to augment antigen-specific spheroid disruption and killing.

**Fig. 2:**
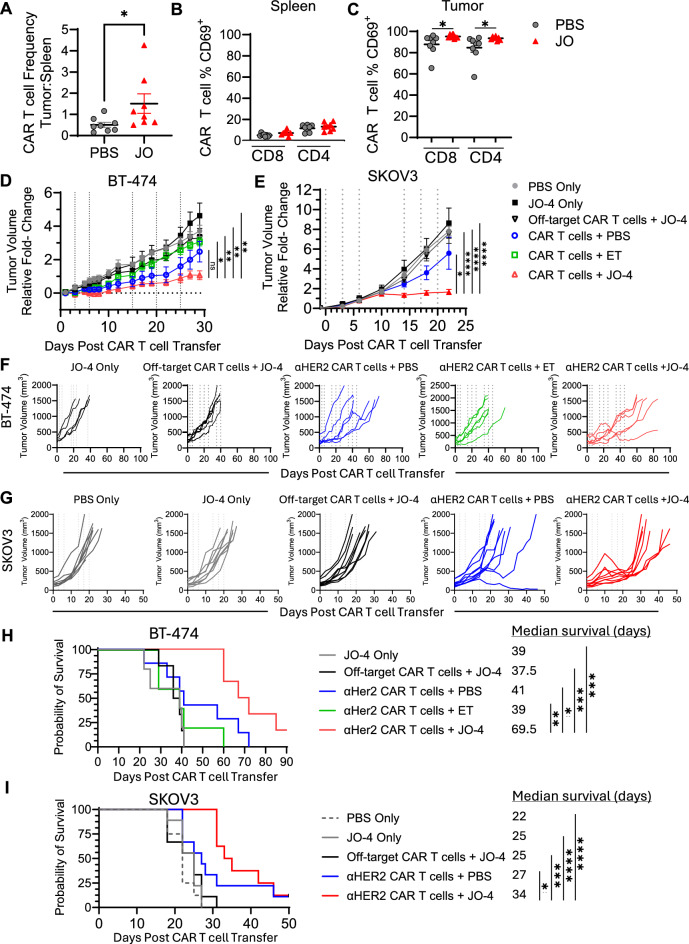
JO-4 treatment enhances CAR T cell-mediated tumor control of solid tumors. BT-474 or SKOV3 tumor cells were implanted into the mammary fat pad or hind flank of female NSG mice respectively (n=5- 6/group BT-474; 8-9 for SKOV3). Once tumors reached an average volume of 250 mm^3^ mice were randomly re-distributed into groups with equal mean tumor volumes. On treatment day 0 off-target or αHER2 CAR T cells, were adoptively transferred i.v.. Following CAR T cell transfer, mice were immediately treated with 2 mg/kg JO-4, PBS, or endotoxin (ET) control (BT-474 only), with repeated treatments indicated by vertical dashed lines. (**A**) Ratio of αHER2 CAR T cell frequency of total viable cells in tumors vs spleens of BT-474 mice on D7 post-CAR T cell transfer. (**B**-**C**) Frequency of activated αHER2 CAR T cells in the spleen vs tumor as determined by CD69-positive expression. (**D**-**E**) Average fold-change in tumor volume of mice bearing (**D**) BT-474, and (E) SKOV3 tumors. (**F**-**G**) Individual tumor volumes. Each line represents an individual mouse. (**H**-**I**) Kaplan-Meier probability of survival, and median survival. Statistics ** = p< 0.01, **** = p<0.0001 as determined by one way ANOVA and Tukey’s multiple comparisons test, or Logrank (Mantel-Cox) test for survival curves.

To examine the impact of JO-4 treatment on CAR T cell distribution and activation *in vivo,* CAR T cells from BT-474 tumors and spleens were analyzed 7-days post-CAR T cell transfer +/- JO-4. JO-4 treatment significantly increased the frequency of αHER2 CAR T cells in tumors compared to spleens (Fig. 2A), and also led to a significant increase in CD4 and CD8 CAR T cell activation in tumors, but not spleens (Fig. 2B, C).

To examine the efficacy of systemic JO-4 treatment in *in vivo* models for breast and ovarian cancers, BT-474 or SKOV3 tumors were established in the mammary fat pad, or flanks, respectively, of NSG mice. Normalized groups of tumor-bearing mice received a suboptimal dose of CAR T cells identified in previous studies. Mice were subsequently treated with controls or JO-4. Additional control groups received either a matched dose of off-target CAR T cells + JO-4 as a control for potential allogeneic anti-tumor responses, or no CAR T cells and PBS or JO-4 to serve as true negative and JO-4 monotherapy controls, respectively. Prolonged tumor control was only observed in mice that received both αHER2 CAR T cells and JO-4 treatments in both the BT-474 and SKOV3 models (Fig. 2D, G). JO-4 treatment led to a significant increase in median survival in αHER2 CAR T cell recipient groups that received ET or PBS, and all other control groups. In contrast, αHER2 CAR T cell recipient groups treated with ET or PBS did not display enhanced survival over off-target CAR T cell, or vehicle control-only treatments (Fig. 2H, I). JO-4 treatment also was well tolerated as treated animals did not lose weight (Supplementary Figure 1).

## Discussion

The collective data reveal that JO-4 combination treatments can enhance CAR T cell therapy by facilitating tumor infiltration, disruption, and cytotoxicity in an antigen-specific manner. This is consistent with previous findings that JO-4 treatment increases access to HER2 on the surface of tumor cells^15^. Trastuzumab-based CAR-T cells efficiently lyse HER2-positive cancer cells^18^, however, not without concern as αHER2 CAR T cells can mediate off-target recognition of normal cells that constitutively express HER2 and result in multi-organ failure^19^. Limiting off-target cell toxicity and improving the therapeutic efficacy of αHER2 CAR T cells to achieve clinical utility is a critical development. Future studies in human-DSG2-expressing models will provide additional insight into the impact of JO-4 treatment on CAR T cell biodistribution.

The addition of JO-4 treatments to CAR T cell infusion enhanced efficacy in BT-474 and SKOV3 tumor models suggest that a similar strategy could permit lower CAR T cell infusion numbers and improved tumor targeting in clinical situations. This has further implications for a reduced need for *ex vivo* expansion of CAR T cells thus reducing the manufacturing time of CAR T cell products and potential for loss of functionality associated with extensive *ex vivo* expansion regimens^20^. The enhanced CAR T cell activation in JO-4 treated tumors suggests that JO-4 treatment could also enhance responsiveness to immune checkpoint therapies.

The data demonstrate the potential of JO-4 treatment to increase responsiveness to CAR T cell immunotherapy against solid tumors.

## Materials and methods

### Cell lines and reagents

#### Tumor cell lines

BT-474 (human breast carcinoma, RRID:CVCL_0179) and SKOV3 (ovarian adenocarcinoma RRID:CVCL-0532) cells were purchased from American Type Culture Collection (ATCC). BT-474 cells were cultured in complete RPMI (Gibco RPMI 1640 + 1% penicillin and streptomycin, and 10% fetal bovine serum (FBS). SKOV3 cells were cultured in McCoy’s 5A medium containing 1% penicillin and streptomycin, and 10% FBS.

To monitor and quantify cells via live-cell imaging, BT-474 cells were transduced to express the red fluorescent protein mKate2 with Incucyte® Nuclight Red lentiviral particles (Sartorius) to generate BT-474-Nuclight Red (BT-474-NLR) cells. Transduced cells were enriched by puromycin selection (1 μg/ml) until >98% of cells were mKate2^+^.

#### CAR T cells

To generate Anti-HER2/Neu (αHer2 CAR) and off-target control (αCD20 CAR) constructs, the VH and VL domains from trastuzumab and rituximab (respectively) were cloned upstream of the CD8 α hinge and transmembrane domains, 4-1BB intracellular domain, CD3 ζ intracellular domain, and eGFP into a lentivirus transfer plasmid and VSV-G pseudotyped lentivirus were produced with a 2^nd^ generation self-inactivating (SIN) system as previously described^21^. Cryopreserved healthy human donor CD3^+^ T cells were thawed into TexMACS™ medium (Miltenyi) supplemented with 5% Human AB serum (Valley Medical), 10 mM neutralized N-acetyl L-Cysteine, β-mercaptoethanol, and 45 IU/ml recombinant IL-2 (PeproTech) and incubated at 37 °C in the presence of 5% CO_2._ After overnight activation with Dynabeads™ Human T-Activator CD3/CD28 (ThermoFisher) the appropriate lentivirus was added. Dynabeads were magnetically removed after three days, and transduction efficiency assessed by flow cytometric detection of eGFP signal. Transduced and non-transduced cell growth was monitored daily, and the cultures expanded with fresh media as needed to maintain cell health. Nine days after transduction, cells were cryopreserved in CryoStor® CS-10 (Biolife Solutions).

Effector cells were defined as CAR^+^ T cells and were calculated by total T cell count × CAR T transduction frequency as determined by eGFP-positivity. CAR transduction efficiency was 89-90%, 93-94%, and 91% for both off-target and anti-HER2 CAR T cells from three separate donors, with CD4 frequencies between 40-50% and CD8 T cell frequencies between 48-56%. CAR intensity was comparable between donors.

#### JO-4

Recombinant JO-4 was produced in *E. coli* Rosetta BL21 (DE3) (EMD Millipore, Darmstadt, Germany) as previously described^22^. Stock JO-4 was stored at a concentration of 0.8 mg/ml in PBS.

### Tumor spheroid formation and treatment

#### Small tumor spheroids

Tumor cells were plated on an AggreWell™ 400 (Stemcell Technologies) plate and prepared as recommended by manufacturer (2.5 × 10^5^ cells per 1,200 microwells). Tumor spheroids were formed over 72-96 hours before use. Loose tumor cells and small spheroids were removed prior to use by straining samples through a 40 µm cell strainer and discarding the flow-through. Approximately 40-50 individual spheroids were plated per-well in 96-well plates immediately prior to use. Experiments included five technical replicates.

#### Large tumor spheroids

BT-474 tumor spheroids were generated by adding 1 × 10^4^ cells per well in ultra low attachment (ULA) 96-well round bottom plates (Corning). Spheroids were formed over 72-96 hours prior to use, with a single tumor spheroid ~1000 µm in diameter formed in each well. Typical spheroid formation was verified for each spheroid prior to use in assays.

#### JO-4 treatment *in vitro*

JO-4 was diluted in matched cell culture media and added to cells to achieve indicated final concentrations. Tumor cells were incubated with JO-4 for 1, or 4, hours (specified in figure legends) prior to CAR T cell addition. Volume of JO-4 or PBS control added to culture wells was < 10% of the total volume per-well. Mice were dosed with 2 mg/kg JO-4 by i.v. injection.

#### CAR T cell spheroid infiltration

Large BT-474 tumor spheroids were generated in a 96-well ultra-low attachment plate. Spheroids were treated with JO-4 at indicated concentrations, or PBS, for four hours prior to CAR T cell addition at a 4:1 effector:tumor ratio. Twenty-four hours post-addition of eGFP-expressing HER2 CAR T cells, tumor spheroids were rinsed with 25 ml of PBS over a 70 µm cell strainer and re-plated for imaging using a KEYENCE BZ-X800 All-in-one fluorescent microscope. All images were taken with matched exposure times and any post-acquisitional modifications (brightness/contrast) were applied universally to all images. Following live-cell imaging, spheroids were treated with 0.25% Trypsin-EDTA for 3 minutes, then gently disrupted through a 40 µm cell strainer µm cell strainer. Single cell suspensions were counted, stained with fluorescent antibodies against CD3ε and viability dye, and acquired via flow cytometry. Total CD3ε+ GFP+ CAR T cells were calculated using the formula (CAR+ T cell frequency of total viable cells × total viable cell count), for each tumor spheroid.

#### Spheroid integrity

Visual spheroid integrity was evaluated over time via images captured on an SX5 Incucyte® real-time cell analyzer.

#### Spheroid cytotoxicity assay

T cells were added to spheroids were added at a 2.5:1 CAR T to tumor cell ratio, or matched total number of non-transduced T cells. SX5 Incucyte with cell-by-cell analysis and Spheroid Analysis software module and analysis using Incucyte Analysis (Sartorius). Untreated controls were used as 0% baseline for cytotoxicity. T cell-only, and tumor cells treated with 1% TritonX, were used as positive controls for tumor cell death/ complete tumor cell killing.

#### Flow cytometry 

Single cell suspensions were stained with Zombie™ fixable viability dye (Biolegend) in PBS, washed, then stained with fluorescently labeled antibodies against CD3 (OKT3), CD4 (RPA-T4), CD8 (RPA-T8), and CD69 (FN50) all from (Biolegend) diluted in PBS containing 1% BSA. Samples were acquired on a BD FACSymphony™ A3 five-laser cytometer using FACSDiva™ acquisition software and analyzed using FlowJo™ V10 (Becton and Dickinson).

#### Mice and tumor models

All animal studies were approved by the University of Washington Department of Comparative Medicine Institutional Animal Care and Use Committee. The facility where animal studies were conducted is accredited by the Association for Assessment and Accreditation of Laboratory Animal Care, International, and follows guidelines set forth by the Guide for the Care and Use of Laboratory Animals, National Research Council, 2011. NOD.Cg-Prkdcscid Il2rgtm1Wjl/SzJ (NSG, Strain #:005557) mice were purchased from Jackson Labs and housed in the University of Washington vivarium. At experimental endpoints, mice were humanely euthanized with 5% CO2 followed by cervical dislocation. Experimental procedures were conducted, and methods reported in accordance with ARRIVE guidelines 2.0 (National Centre for the Replacement Refinement & Reduction of Animals in Research).

### Tumor inoculation and treatments

1 × 10^7^ BT-474 tumors were implanted in the mammary fat pad, or 1 × 10^6^ SKOV3 tumor cells in the hind flank, were mixed with Matrigel™ basement matrix (Corning) at a 1:1 ratio and injected in a 100 μl total volume. Once tumors reached volumes of approximately 250 mm^3^ (~45 d post-tumor implantation), mice were distributed into groups with normalized mean tumor volumes then randomly assigned to treatment groups (RANDBETWEEN, Microsoft Excel). Tumor-bearing mice groups designated for CAR T cell adoptive transfer were intravenously injected with a single dose of either off-target αCD20 or tumor-antigen specific αHER2 CAR T cells. Groups were then subsequently treated by intravenous injection of either PBS, endotoxin (ET) control (matched to ET levels measured in JO-4, for BT-474 experiment only) or JO-4 at 2 mg/kg. Additional treatments were administered on 15, 20, 25, 37,40 and 43 (BT-474) or on days 3, 6, 14, 17 and 20. Tumors were measured every 2-3 days via digital caliper with treatments blinded. CAR T cells from unique healthy donors were used for BT-474 (Donor 1) and SKOV3 (Donor 2). Data are representative of two independent experiments from each tumor type using CAR T cells from distinct donors in repeat experiments. Tumor volume was calculated using the formula V = 0.5 × L × W2, where V is the tumor volume, L is the tumor length, and W is the tumor width. Survival criteria was a tumor volume of >1500 mm^3^ or a > 20% loss in body weight (not observed in any mice in this study).

#### Endotoxin standard

E-Toxate, a preparation of E. coli 055:B5 lipopolysaccharide (Sigma-Aldrich), was diluted in PBS as a vehicle control with matched levels of endotoxin to recombinant JO-4 protein^17^.

#### Software and statistical analysis

Statistical analysis was performed using Graphpad Prism. For *in vitro* assays, 3-5 technical replicates were used in all experiments. Error bars represent ±SEM. * = p<0.05, ** = p< 0.01, *** = p <0.001, **** = p<0.0001 as determined by one way ANOVA and Tukey’s multiple comparisons test. Kaplan-Meier probability of survival plots were used to determine probability and median survival with Logrank (Mantel-Cox) Test used to determine statistical significance between paired groups.

#### Shared resources

This research was supported by the Cellular Imaging Shared Resource RRID:SCR_022609, and Flow Cytometry Shared Resource, RRID:SCR_022613 of the Fred Hutch/University of Washington/Seattle Children’s Cancer Consortium (P30 CA015704).

This study was funded by Astellas Inc.

## Supplementary Information

Supplementary Information.

## Data Availability

The data that support the findings of this study are included in the figures of this manuscript. Raw data files are available from the authors upon reasonable request and with permission of Xyphos, an Astellas company.
