# Supplementary material for: Junction opener enables CAR T cell treatment of solid tumors
Source: Sci Rep. 2026 Mar 7;16:12529. doi: 10.1038/s41598-026-43093-8 (PMC13086946; doi:10.1038/s41598-026-43093-8)
Supplement: Supplementary file 1 — Supplementary Information. [file 41598_2026_43093_MOESM1_ESM.pdf]

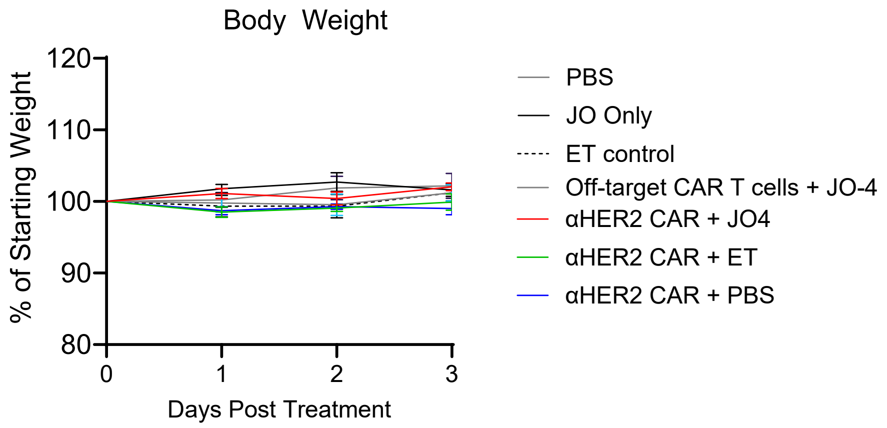

Supplemental Figure 1.
BT-474 tumors were established in the mammary fat pad of NSG mice. Off-target or αHER2 CAR T cells, were adoptively transferred i.v.. Following CAR T cell transfer, mice were immediately treated with 2 mg/kg JO-4, PBS, or endotoxin (ET) control. Percent of pre-treatment body weight are shown ± SEM.
